# Evaluation of an innovative multi-cancer early detection test: high sensitivity and specificity in differentiating cancer, inflammatory conditions, and healthy individuals

**DOI:** 10.3389/fonc.2025.1520869

**Published:** 2025-03-06

**Authors:** Nike Walter, Jörg Groth, Berthold von und zu Zwerger

**Affiliations:** ^1^ University Hospital Regensburg, Regensburg, Germany; ^2^ DRK Hospital Neuwied, Neuwied, Germany; ^3^ Practice for Naturopathy, Garmisch-Partenkirchen, Germany

**Keywords:** multi-cancer early detection, liquid biopsy, diagnostic, cancer screening, plasma protein test

## Abstract

**Background:**

Cancer is a leading cause of death worldwide, with early detection crucial for effective treatment. Traditional diagnostic methods, such as imaging and biopsies, are often limited by invasiveness, cost, and sensitivity. Blood-based multi-cancer early detection (MCED) tests offer a less invasive and potentially more comprehensive approach. Recently, a novel screening tool, the Carcimun^®^ test was reported, detecting conformational changes in plasma proteins through optical extinction measurements. This study evaluates the Carcimun^®^ test’s performance, including participants with inflammatory conditions.

**Methods:**

This prospective, single-blinded study included 172 participants: 80 healthy volunteers, 64 cancer patients (various types), and 28 individuals with inflammatory conditions (fibrosis, sarcoidosis, pneumonia) or benign tumors. Plasma samples were analyzed using the Carcimun^®^ test. Sensitivity, specificity, positive predictive value (PPV), and negative predictive value (NPV) were calculated.

**Results:**

Mean extinction values were significantly higher in cancer patients (315.1) compared to healthy individuals (23.9) and those with inflammatory conditions (62.7) (p<0.001). The Carcimun^®^ test distinguished these groups with high accuracy (95.4%), sensitivity (90.6%), and specificity (98.2%). Significant differences were found between healthy participants and cancer patients (p<0.001), and between cancer patients and those with inflammation (p<0.001).

**Conclusion:**

The Carcimun^®^ test achieved high accuracy, sensitivity, and specificity, effectively identifying cancer patients while minimizing false positives and negatives. By including participants with inflammatory conditions, we addressed a significant limitation of previous studies, demonstrating the test’s robustness in real-world clinical scenarios. These findings underscore the potential of the Carcimun^®^ test as a valuable tool for early cancer detection and screening.

## Introduction

Cancer remains one of the leading causes of death worldwide, with an estimated 10 million deaths in 2022 alone. The data indicates that about one in five people will be diagnosed with cancer during their lifetime, with approximately one in nine men and one in twelve women succumbing to the disease (1). The ability to detect cancer at an early stage, when treatment is more likely to be successful, is crucial for reducing cancer-related mortality and improving patient outcomes (2). Traditional methods for cancer detection include imaging techniques, tissue biopsies, and specific screening tests for individual cancers. However, these conventional approaches, although constantly advancing, often face significant limitations, including invasiveness, high costs, and limited sensitivity, especially for cancers that are asymptomatic or located in hard-to-reach areas (3, 4). In recent years, the field of oncology has seen a paradigm shift with the advent of blood-based multi-cancer early detection (MCED) tests. These innovative tests are designed to detect multiple types of cancer by identifying and analyzing molecular signals associated with malignancy (5). Several blood-based MCED tests are currently in development and undergoing clinical evaluation. Among the most studied biomarkers for liquid biopsy are circulating tumor DNA (ctDNA) (6–9). These DNA fragments can carry genetic mutations, methylation patterns, and other alterations characteristic. For example, GRAIL’s Galleri test uses a targeted methylation sequencing approach to detect over 50 types of cancer from a single blood draw (5). However, one primary challenge is the low abundance of ctDNA in the bloodstream, especially in early-stage cancers, which can result in low sensitivity and a higher likelihood of false negatives. This issue is compounded by the fact that ctDNA must be distinguished from the much larger background of normal cell-free DNA, necessitating highly sensitive and specific analytical techniques. Additionally, ctDNA can exhibit considerable heterogeneity across different cancer types and even within different tumors of the same type. This variability can complicate the development of a universal detection method, as different cancers may release varying quantities and types of ctDNA, potentially leading to inconsistent detection rates (10). Furthermore, the costs associated with ctDNA analysis can be high, particularly when using advanced sequencing technologies (11). Recently, a novel pancancer test (Carcimun^®^) utilizing a different approach was reported. This test detects conformational changes in plasma proteins through optical extinction measurements, offering a more universal marker for general malignancy and acute inflammation. The feasibility and performance of the Carcimun^®^ test was assessed in a prospective study with 137 healthy volunteers and 170 cancer patients (n=16 different entities), achieving high accuracy (90.0%), sensitivity (88.8%), and specificity (91.2%). These findings suggest the potential of the Carcimun^®^ test to advance the early diagnosis and screening of various cancers, however, a major limitation included the drop out criteria of participants with elevated inflammatory markers (12).

Therefore, this study aims at (i) evaluating the performance of the Carcimun^®^ test in a more inclusive cohort that incorporates participants with inflammatory conditions and (ii) validate the test’s overall accuracy, sensitivity, and specificity. By including participants with inflammatory conditions, this study seeks to provide a comprehensive evaluation of the Carcimun^®^ test’s utility in real-world clinical scenarios, thereby enhancing its applicability for early cancer diagnosis and screening.

## Methods

This prospective study included a total of 172 participants. Participants were recruited based on their clinical availability during the study period. The cohort composition enabled in initial assessment of the Carcimun^®^ test’s diagnostic performance across a range of clinical presentations. Testing with the Carcimun^®^ test was performed as part of a general screening process, irrespective of whether malignancy was initially suspected. The diagnostic status of each patient was determined subsequently, based on standard clinical protocols. Cancer diagnoses were confirmed in n=64 cases using imaging techniques and/or histopathological evaluation ensuring accurate classification. All cancer cases included in the study were classified as stages I–III at the time of diagnosis. Tumors included both symptomatic and asymptomatic cases, reflecting a broad spectrum of clinical presentations. The majority of samples were referred from a lung specialist as part of a broader cancer screening program. From these, n=26 participants were ultimately diagnosed with fibrosis (n=9), sarcoidosis (n=9), or pneumonia (n=8) following diagnostic evaluations. The cohort further comprised two patients, which were initially suspected of malignancy but later confirmed to have benign tumors.

All participants underwent blood sampling and the extinction values were measured using the Carcimun^®^ test as described previously (12). Specifically, samples were prepared by initially adding 70 µl of 0.9% NaCl solution to the reaction vessel, followed by 26 µl of blood plasma, resulting in a total volume of 96 µl with a final NaCl concentration of 0.9%. Subsequently, 40 µl of distilled water (aqua dest.) was added, increasing the volume to 136 µl and adjusting the NaCl concentration to 0.63%. The mixture was incubated at 37°C for 5 minutes to achieve thermal equilibration. After incubation, a blank measurement was recorded at 340 nm to establish a baseline. Following this, 80 µl of 0.4% acetic acid (AA) solution (containing 0.81% NaCl) was added, resulting in a final volume of 216 µl with 0.69% NaCl and 0.148% acetic acid. The final absorbance measurement was performed at 340 nm using the Indiko™ Clinical Chemistry Analyzer (Thermo Fisher Scientific, Waltham, MA, USA). To ensure the integrity of the results, all measurements were performed in a blinded manner, meaning that the personnel conducting the extinction value measurements were unaware of the clinical or diagnostic status of the samples.

The test’s performance metrics, including sensitivity, specificity, positive predictive value (PPV), and negative predictive value (NPV), were calculated using a previously defined cut-off value of 120 to differentiate between healthy and cancer subjects (12). This threshold was determined in an independent cohort from a prior study, which included n=241 non-pathologic and n=114 pathologic reference samples (12). The cut-off was optimized using statistical methods including receiver operating characteristic (ROC) curve analysis and the Youden Index.

This study was conducted in accordance with the Declaration of Helsinki. Ethical approval was obtained from the Institutional Review Board of the State Medical Association Rheinland-Pfalz with approval number 837.262.13 (8947-F). Written informed consent was obtained from all participants prior to their inclusion in the study.

Descriptive and statistical data analysis was performed using the IBM SPSS Statistics software (version 28.0, IBM Corp., Armonk, NY, USA). Continuous parameters were presented as means ± standard deviation (SD). One-way ANOVA with Tukey- and Games–Howell *post-hoc* test was conducted after ensuring homogeneity of variances using Levene’s test and normal distribution by Shapiro–Wilk test. For all tests, *p*-values ≤ 0.05 were considered statistically significant.

## Results

Participants were divided into three categories: (i) healthy participants, (ii) patients with a verified malignancy, and (iii) patients with a verified inflammatory diagnosis (fibrosis, sarcoidosis, pneumonia) n=26 or a benign tumor (n=2) (**Table 1**). Group two consisted of patients with pancreatic cancer n=5, bile duct cancer n=5, liver metastasis n=5, esophageal cancer n=5, stomach cancer n=5, gastrointestinal stromal tumor (GIST) n=5, peritoneal cancer n=5, colorectal cancer n=10, and lung cancer n=19 in the stages I-III.

**Table 1:** Patients diagnosed with inflammatory conditions or benign tumor.

	Healthy subjects n= 80	Patients diagnosed with cancer n=64	Patients diagnosed with inflammatory conditions and benign tumor n=28	Statistical significance
Age (years)	49.1 ± 5.8	54.8 ± 6.3	51.3 ± 7.4	p= 0.807
Sex (female/male)	37/43	28/36	12/16	p= 0.768
Mean extinction Carcimun-test	24.3± 23.9	218.0 ± 188.9	46.7 ± 22.7	p< 0.001

**Figure 1:**
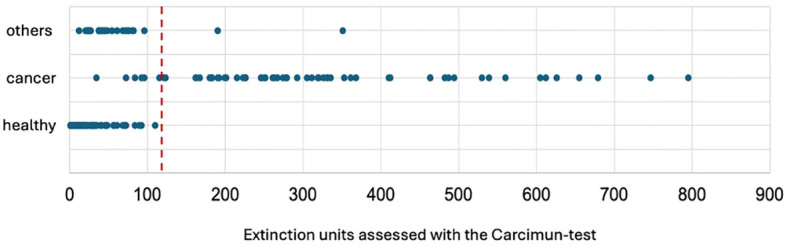
Extinction units assessed with the Carcimun® test shown for the three groups of participants. The extinction threshold of 120 U to differentiate cancer patients is shown as the red dotted line.

The extinction values ranged from 1-110 (mean 23.9) in group 1 (healthy), from 12-351 (mean 62.7) in group 3 (other), and from 34-795 (mean 315.1) in group 2 (malignancy) (**Figure 1**). Thus, the comparison of the mean extinction values revealed a 5.0-fold increase comparing cancer patients with healthy individuals. The Carcimun value differed statistically significant for the three groups (one way ANOVA: F=128.65, p< 0.001) with an effect size of η²=0.60. Tukey *post-hoc* analysis revealed a significant difference between healthy patients and patients diagnosed with cancer (291.13, 95%-CI: 246.86 to 335.39, p<0.001]), as well as between patients diagnosed with cancer and patients with inflammatory processes (252.39, 95%-CI: 192.58 to 312.19, p<0.001]). There was no statistically significant difference when comparing healthy patients and patients with inflammation (38.74, 95%-CI: -19.21 to 96.70, p=0.257).

**Figure 2:**
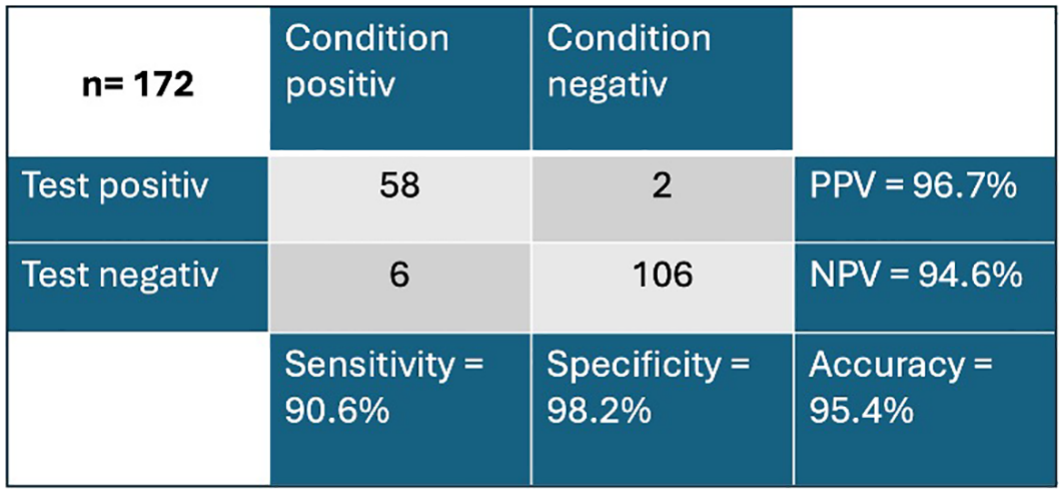
Overview of true negative and true positive values of the Carcimun® test PPV, positive predictive value; NPV, negative predictive value.

In previous studies, an extinction value of 120 had been defined as a cut-off to differentiate between healthy subjects and cancer subjects (<120) (12). Applying this cut-off, the Carcimun^®^ test achieved high levels of accuracy (95.4%), sensitivity (90.6%), and specificity (98.2%) in this cohort. The positive and negative predictive values resulted in 96.7% and 94.6%, respectively (**Figure 2**). All participants in the study group one were correctly identified as healthy. False positive results were observed in two cases, one patient diagnosed with an acute pneumonia and one with severe sarcoidosis. False negative results occurred in patients with lung cancer (3/19), stomach cancer (2/5) and GIST (1/5).

## Discussion

The study demonstrated that the Carcimun^®^ test effectively differentiates between healthy individuals, patients with verified malignancies, and those with inflammatory or benign conditions. The Carcimun^®^ test demonstrated high efficacy with an accuracy of 95.4%, sensitivity of 90.6%, and specificity of 98.2%. These metrics are competitive with, and in some cases superior to, DNA-based tests.

Several other studies have been dedicated to blood-based cancer diagnostic tests, providing a context for evaluating the Carcimun^®^ test ‘s performance. For instance, the Galleri test (GRAIL, Menlo Park, California) has been one of the most discussed innovations in the MCED field. It has been evaluated in several pivotal studies. In the Circulating Cell-free Genome Atlas (CCGA) substudy (13), which involved a large case-control cohort of 4,077 participants (2,823 cancer patients), Galleri reported a n overall sensitivity of only 51.5% (95% CI 49.6% to 53.3%). Despite its high specificity of 99.5% (95% CI 99.0% to 99.8%), this level of sensitivity implies that nearly half of the cancer cases could be missed, particularly those in the early stages where intervention could be most effective. Furthermore, the PATHFINDER study (14) expanded on Galleri’s assessment by including 6,369 participants, split into elevated and non-elevated cancer risk cohorts. The sensitivity in this broader, more representative population dropped significantly to 20.8% (95% CI 14.0% to 29.2%), highlighting the limitations of the test and the challenges associated with ctDNA detection (10, 15). Additionally, in the SYMPLIFY study, which included 5,461 symptomatic individuals, Galleri’s sensitivity was reported at 66.3% (95% CI 61.2% to 71.1%) with a specificity of 98.4% (95% CI 98.1% to 98.8%), representing an improvement (16). Also, CancerSEEK (Exact Sciences, Madison, Wisconsin), a liquid biopsy test, which works by analyzing a combination of genetic mutations in ctDNA and protein biomarkers that are commonly associated with cancer, has been evaluated. One notable case-control study involved 1,817 participants, including 1,005 patients with various types of cancer and 812 healthy controls. In this study, CancerSEEK demonstrated a sensitivity of 62.3% (95% CI 59.3% to 65.3%) and a high specificity of 99.1% (95% CI 98.5% to 99.8%) (17). The DETECT-A study further assessed CancerSEEK’s performance in a real-world, prospective cohort of 9,911 women. However, the sensitivity in this larger and more diverse cohort dropped significantly to 27.1% (95% CI 18.5% to 37.1%). This decrease in sensitivity was particularly pronounced in early-stage cancers, with the test detecting only 12.7% (95% CI 6.6% to 23.1%) of stage I-II cancers (18). Another MCED test combining ctDNA analysis with the measurement of specific protein biomarkers is the Cancer Differentiation Analysis (CDA) test (AnPac Bio, Shanghai, China). It was evaluated in a prospective population-based cohort study in China, which followed 1,957 participants over a median of 15 months. The study reported a sensitivity of 40% (95% CI 12.2% to 73.8%) and a high specificity of 97.6% (95% CI 96.8% to 98.2%) (19). In contrast, the TruCheck test TruCheck™ (Datar Cancer Genetics, Beyreuth, Germany) demonstrated high sensitivity in both the RESOLUTE and TrueBlood studies conducted in India. The technology underlying TruCheck is based on detecting circulating tumor cells (CTCs) and ctDNA in the blood. The RESOLUTE study, which included 6,884 participants, reported a sensitivity of 90% (95% CI 55.5% to 99.7%) and a specificity of 96.4% (95% CI 95.9% to 96.8%) (20). Similarly, the TruBlood study involved 9,920 participants and found a sensitivity of 93% (20), which is comparable to the presented figures of the Carcimun^®^ test. Further, the SPOT-MAS test (Gene Solutions, Ho Chi Minh City, Vietnam) focuses on detecting specific serum protein biomarkers that are often elevated in the presence of cancer. It was evaluated in the K-DETEK cohort study in Vietnam, involved 2,792 participants aged over 40 who had no clinical suspicion or history of cancer. Despite its high specificity of 99.9% (95% CI 99.6% to 100%), which suggests the test is very effective at correctly identifying individuals without cancer, its sensitivity was limited due to the small number of positive cases detected. Only 10 participants (0.36%) tested positive, and of these, six were confirmed to have cancer, leading to a positive predictive value (PPV) of 60% (95% CI 26.2% to 87.8%) (21). This high specificity indicates that SPOT-MAS is reliable in ruling out non-cancer cases, but the limited sensitivity suggests the test may miss a significant number of actual cancer cases, particularly in a screening setting. Finally, the AICS test (AminoIndex Cancer Screening, Ajinomoto, Japan) represents a novel approach using artificial intelligens (AI) algorithms to analyze plasma-free amino acid profiles in blood (22). The study followed participants for up to 6.2 years, showing varying sensitivity depending on the six different cancer type analyzed. For example, the sensitivity for detecting breast cancer was 29.0% while for colorectal cancer, it was 28.6%. The AICS test shows promise due to its innovative use of AI, but the variability in sensitivity across different cancer types underscores the need for further refinement and rather than providing a binary results, the test ranks individuals on the probability of having each of the cancers tested (23).

MCED technology, such as the Carcimun^®^ test, has the potential to significantly improve oncological screening by simplifying and streamlining the diagnostic journey for patients while optimizing healthcare resource allocation. Its high sensitivity and specificity make it a promising candidate as a first-line screening tool in routine health check-ups or for individuals presenting with mild, non-specific symptoms (e.g., fatigue, weight loss, or persistent inflammation). By providing an early indication of whether malignancy is present, the Carcimun^®^ test could facilitate earlier intervention, which is critical for improving cancer outcomes (24). As a minimally invasive, blood-based test, the Carcimun^®^ test can be easily implemented in routine clinical settings, including primary care clinics, community health centers, or screening programs. This decentralized approach ensures broader accessibility, particularly in resource-limited settings or regions with inadequate access to specialized oncology services. In these scenarios, the Carcimun^®^ test could address significant gaps in early cancer detection and equity of care. Patients with test results below the threshold could be reassured of a low likelihood of malignancy, reducing unnecessary anxiety and follow-up procedures. This could minimize referrals to specialists, alleviate strain on healthcare systems, and reduce the costs associated with imaging, biopsies, and consultations. For patients with test results above the threshold, the Carcimun^®^ test could guide clinicians in prioritizing additional diagnostic procedures, such as imaging (e.g., CT, MRI, or PET scans), to confirm the diagnosis and localize the tumor. This triage approach would help ensure that high-risk patients receive timely and appropriate care, reducing diagnostic delays that could worsen prognosis. Furthermore, Carcimun^®^ test results could complement imaging and histopathological methods, providing a more comprehensive diagnostic picture to refine the diagnostic pathway. For instance, combining the test with imaging could improve diagnostic accuracy by compensating for false negatives or positives that may arise with either modality when used in isolation. Beyond its clinical utility, the Carcimun^®^ test could also bring substantial economic benefits (25). By accurately identifying low-risk patients at an early stage, the Carcimun^®^ test could decrease the frequency of unnecessary imaging and invasive procedures. For high-risk patients, its ability to triage and focus resources on those most likely to benefit from further diagnostic work-up would optimize healthcare resource utilization. Despite these strengths, several shortcomings and challenges must be critically addressed. A key limitation of the Carcimun^®^ test is its inability to localize tumors. While the test can identify the presence of malignancy, it does not provide information about the anatomical site or the type of cancer. This limitation necessitates additional diagnostic procedures, such as imaging or biopsies, to confirm and localize the malignancy. The reliance on complementary diagnostics reduces the standalone utility of the test and could delay definitive diagnoses in certain settings. To this end, the Carcimun^®^ test should be integrated into a multimodal screening protocol rather than being used as a standalone diagnostic tool. However, real-world implementation poses logistical challenges, such as the need for consistent test quality, training healthcare providers to interpret and act on test results and establishing standardized protocols for follow-up diagnostics. Another significant challenge is the potential for false-positive and false-negative results. False positives could lead to unnecessary anxiety for patients and increased healthcare expenditures due to unwarranted follow-up imaging or biopsies. On the other hand, false negatives are particularly concerning, as they may result in delayed diagnoses and missed opportunities for early intervention, ultimately impacting patient survival (24).

In this study, false negatives were observed in six cases, where the test failed to detect lung cancer (3/19), stomach cancer (2/5), and GIST (1/5). These discrepancies could stem from multiple factors that complicate the detection of cancer-specific biomarkers. One potential explanation is the presence of undocumented, subclinical, non-malignant conditions that create background variability in systemic protein profiles. Such variability could obscure the cancer-specific signals required for accurate detection, highlighting a key challenge in distinguishing subtle tumor biomarkers from broader inflammatory or physiological noise. Different tumors exhibit unique molecular and genetic signatures that impact their ability to release detectable biomarkers into the bloodstream. Some tumors driven by mutations that activate localized signaling pathways may not produce systemic changes significant enough to be captured by blood-based assays. GISTs, for example, are frequently associated with activating mutations in the KIT or PDGFRA genes (26), resulting in constitutive activation of their respective tyrosine kinase receptors. However, these molecular alterations are typically localized to the tumor microenvironment and may not lead to significant changes in systemic protein levels, particularly in smaller or early-stage tumors. This could explain the inability of the Carcimun^®^ test to detect one GIST case in this study. Specific post-translational modifications of proteins, such as glycosylation or phosphorylation may also account for the failure in some cases, altering solubility properties and interfering with the effective precipitation during the assay. False-positive results were observed in two cases: one patient with acute pneumonia and another with severe sarcoidosis. Such conditions are known to induce systemic changes in blood coagulation and fibrinolysis pathways, which can be misinterpreted by the test as cancer-specific signals. For instance, increased levels of plasminogen activator inhibitor-1 (PAI-1), which suppress fibrinolysis, may contribute to clot persistence, yielding elevated test values such as in cancer cases (27). Similarly, inhibited fibrinolytic activity could lead to the accumulation of fibrin degradation products, such as elevated D-dimers, mimicking cancer-associated hypercoagulability. To minimize errors, it is recommended to monitor these hematological and coagulation parameters during testing (28, 29).

### Limitations

Despite the promising results demonstrated by the Carcimun^®^ test, several limitations need to be acknowledged. Firstly, the relatively small sample size, especially within specific cancer subgroups such as pancreatic cancer and GIST, may limit the generalizability of the findings. This limitation underscores the necessity for larger, more diverse cohorts to confirm the robustness and reliability of the test’s performance across different populations. Additionally, while the study included various cancer types, the performance of the Carcimun^®^ test for specific cancers was not evaluated in depth. Since different cancers might present with varying results, further studies focusing on individual cancer types are essential to better understand the test’s efficacy in each context. Also, while cancer cases in the study included stages I–III, subgroup analyses by disease stage were not performed due to the limited sample sizes within each stage, which would reduce statistical power and the reliability of the results. This study is exploratory and focused on validating the overall diagnostic performance of the Carcimun^®^ test. Future studies with larger, stage-stratified cohorts are planned to specifically assess test performance across different cancer stages. Further, the study included participants with inflammatory conditions (fibrosis, sarcoidosis, pneumonia) to address a key limitation of prior studies. However, the range of inflammatory conditions tested was limited, and the test’s performance in other inflammatory diseases or benign conditions remains to be explored in depth. Expanding the scope of inflammatory conditions in future studies and also assessing other markers such as CRP and ESR to evaluate severity of the conditions will be critical to fully assess the test’s specificity. Lastly, the cross-sectional design of the study limits its ability to evaluate the Carcimun^®^ test for monitoring cancer progression or detecting recurrences over time. Longitudinal studies are necessary to validate its utility in these clinical scenarios. In the same stance, follow-up assessments for patients classified as healthy or with inflammatory conditions were not conducted as part of this study. This limitation is acknowledged and highlights the need for future studies incorporating longitudinal follow-up to assess the long-term diagnostic reliability of the Carcimun^®^ test and its capacity to detect conditions that might develop over time.

### Conclusion

The Carcimun^®^ test achieved high accuracy, sensitivity, and specificity, effectively identifying cancer patients while minimizing false positives and negatives. By including participants with inflammatory conditions, we addressed a significant limitation of previous studies, demonstrating the test’s robustness in real-world clinical scenarios. These findings underscore the potential of the Carcimun^®^ test as a valuable tool for early cancer detection and screening.

## Data Availability

The raw data supporting the conclusions of this article will be made available by the authors, without undue reservation.
